# Gauging U.S. Emergency Medical Services Workers' Willingness to Respond to Pandemic Influenza Using a Threat- and Efficacy-Based Assessment Framework

**DOI:** 10.1371/journal.pone.0009856

**Published:** 2010-03-24

**Authors:** Daniel J. Barnett, Roger Levine, Carol B. Thompson, Gamunu U. Wijetunge, Anthony L. Oliver, Melissa A. Bentley, Patrick D. Neubert, Ronald G. Pirrallo, Jonathan M. Links, Ran D. Balicer

**Affiliations:** 1 Johns Hopkins Preparedness and Emergency Response Research Center, Baltimore, Maryland, United States of America; 2 Department of Environmental Health Sciences, Johns Hopkins Bloomberg School of Public Health, Baltimore, Maryland, United States of America; 3 American Institutes for Research, San Mateo, California, United States of America; 4 Department of Biostatistics, Johns Hopkins Bloomberg School of Public Health, Baltimore, Maryland, United States of America; 5 Office of Emergency Medical Services, National Highway Traffic Safety Administration, United States Department of Transportation, Washington, D. C., United States of America; 6 Association of Schools of Public Health, Washington, D. C., United States of America; 7 National Registry of Emergency Medical Technicians, Columbus, Ohio, United States of America; 8 Department of Emergency Medicine, Medical College of Wisconsin, Milwaukee, Wisconsin, United States of America; 9 Milwaukee County Emergency Medical Services, Milwaukee County, Wisconsin, United States of America; 10 Department of Epidemiology, Faculty of Health Sciences, Ben-Gurion University of the Negev, Beer-Sheva, Israel; The University of Melbourne, Australia

## Abstract

**Background:**

Emergency Medical Services workers' willingness to report to duty in an influenza pandemic is essential to healthcare system surge amidst a global threat. Application of Witte's Extended Parallel Process Model (EPPM) has shown utility for revealing influences of perceived threat and efficacy on non-EMS public health providers' willingness to respond in an influenza pandemic. We thus propose using an EPPM-informed assessment of EMS workers' perspectives toward fulfilling their influenza pandemic response roles.

**Methodology/Principal Findings:**

We administered an EPPM-informed snapshot survey about attitudes and beliefs toward pandemic influenza response, to a nationally representative, stratified random sample of 1,537 U.S. EMS workers from May–June 2009 (overall response rate: 49%). Of the 586 respondents who met inclusion criteria (currently active EMS providers in primarily EMS response roles), 12% indicated they would not voluntarily report to duty in a pandemic influenza emergency if asked, 7% if required. A majority (52%) indicated their unwillingness to report to work if risk of disease transmission to family existed. Confidence in personal safety at work (OR = 3.3) and a high threat/high efficacy (“concerned and confident”) EPPM profile (OR = 4.7) distinguished those who were more likely to voluntarily report to duty. Although 96% of EMS workers indicated that they would probably or definitely report to work if they were guaranteed a pandemic influenza vaccine, only 59% had received an influenza immunization in the preceding 12 months.

**Conclusions/Significance:**

EMS workers' response willingness gaps pose a substantial challenge to prehospital surge capacity in an influenza pandemic. “Concerned and confident” EMS workers are more than four times as likely to fulfill pandemic influenza response expectations. Confidence in workplace safety is a positively influential modifier of their response willingness. These findings can inform insights into interventions for enhancing EMS workers' willingness to respond in the face of a global infectious disease threat.

## Introduction

Against a broad array of all-hazards threats, research efforts have increasingly focused on the willingness of a variety of types of healthcare providers to perform their duties in emergency and disaster settings [1]–[9]. This expanding body of evidence has revealed that rates of *willingness* to respond (an attitudinal domain) frequently differ substantially from rates of *ability* to respond (a skill-and knowledge-based domain) – even for the same cohort within the same situational context [1], [2]. Such findings have also underscored that response willingness is scenario-specific [1], [2] and influenced by a variety of peripheral risk perception factors apart from the actual hazard [3], [4]. An integrative review of the willingness-to-respond research literature has highlighted a need for enhanced use of conceptual and theoretical frameworks to predict new phenomena and generate relevant hypotheses for this field of inquiry [10]. Recent research has illustrated how hazard-specific perceptions of threat (“concern”) and efficacy (“confidence”) respectively modify response willingness among public health workers [4], based on direct application of a fear appeal-based behavioral model – Witte's Extended Parallel Process Model (EPPM) – that describes adaptive behavior in the face of unknown risk Specifically, the EPPM describes how messages regarding adaptive protective behaviors in risk contexts will be apt to elicit these desired behaviors if the message recipients: 1) perceive the message's threat component as worthy of attention (threat appraisal); and 2) consider the message's target behavior to be achievable and beneficial (efficacy appraisal) [11].

The willingness of healthcare providers to fulfill emergency response expectations is particularly salient in an influenza pandemic – an occurrence which exerts a considerable human resource burden on the healthcare infrastructure [12]. While the course of an influenza pandemic is often unpredictable, the epidemiologic pattern of the currently circulating pandemic H1N1 2009 influenza A has thus far appeared more akin to the relatively milder 1957–58 “Asian” influenza (H2N2) pandemic than to the severe 1918–19 “Spanish” influenza pandemic [13]. Still, the current pandemic influenza viral strain has been shown to cause serious complications and mortality among children and healthy young adults [14] – a characteristic which increases its associated dread and makes it markedly different from seasonal influenza. Health authorities in the northern hemisphere are preparing for a “second wave” that may be even more severe and disseminated than the early summer wave in the northern hemisphere [15]. This second wave may impose a significant burden on all healthcare organizations.

Recent research on willingness to respond among public health emergency preparedness system providers has indicated that nearly 1 in 6 local public health department workers would not be willing to report to duty in an influenza pandemic “regardless of severity” [4]. Additional studies have uncovered marked gaps in pandemic influenza response willingness among a cohort of hospital-based healthcare providers in the US [7], and of hospital- and community-based healthcare providers within the UK [12]. Collectively, these findings point toward the need for enhanced understanding of pandemic influenza response attitudes and beliefs among Emergency Medical Services (EMS) personnel, whose failure to report to work (for reasons other than illness) could further compromise already-limited surge capacity [16].

EMS workers play an integral role within the public health emergency preparedness system [17], and are on the frontlines of response to patients' urgent medical needs throughout an influenza pandemic. EMS personnel typically provide out-of-hospital medical care to patients with perceived urgent needs. Most EMS personnel work in EMS organizations that respond to 9-1-1 emergency calls. Emergency Medical Technicians (EMTs) are trained to respond at a moment's notice to perform skills such as cardiopulmonary resuscitation, lifesaving airway management interventions, and other basic, non-invasive medical interventions. Paramedics are further trained to deliver critical medications and perform fluid resuscitation, perform advanced cardiac life support, and other invasive and pharmacological interventions [18].

Given the essential roles of EMS workers, understanding their response willingness in an influenza pandemic is relevant to both practice and policy. However, significant research gaps have existed to date regarding these workers' attitudes and perceptions toward pandemic influenza response. Moreover, prior studies on healthcare workers' willingness to respond in pandemics have been based on surveys conducted exclusively during interpandemic periods. Through introduction of an EPPM-informed questionnaire into a national longitudinal survey of EMTs and Paramedics from May to June 2009, we thus aim to identify the relative influences of perceived threat and efficacy on EMS workers' response willingness in the face of a pandemic threat, and to uncover additional relevant barriers and facilitators of pandemic influenza response willingness among this cohort.

## Methods

### Ethics Statement

Institutional Review Board (IRB) approval for the 2009 Pandemic Influenza survey was obtained from the American Institutes for Research (AIR). The present study presented no more than minimal risks to participants involved, and participants received a cover letter indicating their rights as study participants with emphasis on voluntary participation. Written consent was not required by the AIR IRB.

### Survey Instrument

The Longitudinal EMT Attributes and Demographics Study (LEADS) is a prospective study of EMS workers conducted annually by the National Registry of EMTs (NREMT), a national EMS certification agency. Support for data analysis is provided by the National Highway Traffic Safety Administration (NHTSA) through the American Institutes for Research (AIR). The LEADS project consists of a longitudinal set of 77 core survey items that annually collects information including demographics, education, finance and health-related topics. Further, the LEADS survey also consists of a snapshot survey, modified each year, which has allowed the collection of data on topics ranging from (but not limited to) disaster preparedness, compensation and benefits, sleep problems, and occupational commitment.

Episodically, a mid-year survey is conducted and mailed to study participants who responded to the most recent LEADS survey. Mid-year surveys are constructed based on recommendations from the LEADS committee, a steering committee of EMS workforce researchers appointed by the NREMT. In January 2009, the LEADS committee developed a pandemic influenza survey in collaboration with the Johns Hopkins Preparedness and Emergency Response Research Center. This survey assessed personal preparedness and willingness to respond to an influenza pandemic among EMS personnel who had responded to the 2008 LEADS survey. Vaccination survey questions were adopted from the Centers for Disease Control and Prevention's (CDC) 2007 National Health Interview Survey. Personal preparedness questions were modeled after the Federal Emergency Management Agency's Public Readiness Index.

Willingness-to-respond questions were modeled from the *Johns Hopkins∼Public Health Infrastructure Response Survey Tool (JH PHIRST)* [4]. The *JH∼PHIRST* instrument is designed to assess respondents' attitudes and beliefs toward public health emergency response, using a 10-point Likert scale (strong agreement to strong disagreement, including a “don't know” option). The *JH∼PHIRST* incorporates aspects of risk perception, threat and efficacy measures from the Extended Parallel Process Model (EPPM) [11], which has been extensively validated in multiple national, cultural, and healthcare contexts [19].

### Study participants

The LEADS Project survey sample was selected from the NREMT database, and comprised a nationally representative sample of EMS personnel in primarily EMS response roles (Emergency Medical Technician – Basics [EMTs] and Emergency Medical Technician – Paramedics [Paramedics]) in the United States. Stratified random sampling of respondents to the 2008 LEADS Survey was used to obtain a representative sample of EMS workers who are stratified on ethnicity (white versus minority), years of EMS national registration (one year or less versus one year or more), and level of EMS certification (EMT or Paramedic). Participants are surveyed annually until they stop responding to requests to complete the questionnaire. Each individual is assigned a statistical weight based on the stratum to which they belong in the year of survey completion. As this population is dynamic from year to year, sampling frames change on an annual basis. Therefore, stratum-specific weights also vary on an annual basis. Non-responder surveys are also mailed each year to better define the non-responding population. Participants in the current study were mailed a survey and a letter of explanation, and were surveyed from May 15, 2009–June 30, 2009.

### Statistical Analysis

The survey responses were merged with respondent demographic information from the 2008 LEADS survey. The 2008 statistical weights were adjusted for differential response rates within each stratum, enabling generalization to the universe of all individuals who were nationally registered EMTs and Paramedics in 2008. Those whose primary role was educator, manager, supervisor, administrator, or other were excluded from the analyses. Prior to analysis, “don't know” responses to the 10-point Likert-scale items were assigned the construct-specific (or attitude/belief-specific) median value of the Likert-scale responses. These responses were then dichotomized into categories of ≤5 (‘positive response’) versus >5 (‘negative response’). Four scenario-specific categories for the Extended Parallel Process Model (EPPM) were also created, based on level of perceived threat and level of perceived efficacy. These categories include: low threat and low efficacy; low threat and high efficacy; high threat and low efficacy; and finally, high threat and high efficacy. Using the Likert-scale responses, the ‘threat’ variable was determined as the product of the respondent's perceived likelihood of the occurrence of the given public health threat and the perceived severity of the event, while the ‘efficacy’ variable was calculated as the product of the participant's perceived ability to perform their duty and their perceived impact on combating the given public health threat. Low and high categories of threat and efficacy were determined by the median values of these two variables, respectively. For example, Likert-scale responses of 2 and 4 for the likelihood and severity statements of the threat dimension give a cross-product score of 8. The median of the threat cross-product for all respondents is 24, which would assign the score of 8 to the high-threat category, below the median. The median of the efficacy cross-products is 5, and a cross-product score of 8 would be assigned to the low-efficacy category, above the median.

Weighted logistic regression analyses were performed to evaluate relationships between the willingness-to-respond attitudes, EPPM threat and efficacy categories, and knowledge about pandemic influenza as outcomes; and EMS professional characteristics and census region designations, and their attitudes and beliefs as predictors. Regression analyses were performed, adjusting for other EMS professional characteristics and their attitudes and beliefs. Missing responses were excluded from the analyses. Characteristics of respondents who had received an influenza immunization in the preceding 12 months were compared with those not receiving an influenza immunization, using chi-squared tests. All analyses were performed using SAS version 9.1 (SAS Inc, Cary, NC).

## Results

Seven hundred and fifty-three (49.0%) of the EMS workers who took the 2008 LEADS survey also responded to this survey. Of these respondents, 586 indicated that they were currently performing EMS work and that their primary EMS role was associated with the provision of clinical EMS Services. The analyses performed were weighted based on the respondent's stratum as defined above for the LEADS project survey, and thus results are provided only in terms of percentages or odds ratios. Table 1 describes the respondents to include a majority of males (66%), older than 35 years of age (63%), less than a bachelors' degree (71%), belonging to a non-fire-based organization (61%), having five or more years of experience (56%), are EMTs (66%), being from or serving a rural area (52%), working as clinicians rather than in fire-suppression (83%), and working for only one EMS organization (68%). The categorization of the EMS characteristics was based on the lack of association observed with finer categorizations in earlier work with health department cohorts using a closely-aligned survey.

**Table 1:** Characteristics of EMS clinical service providers (weighted).

Characteristics	%
Gender:	
Female (vs Male)	34.1
Age:	
>35 years (vs ≤35 years)	63.2
Highest degree:	
At least a bachelor's degree (vs Less than a bachelor's degree)	29.0
Type of organization to which they belong	
Fire-suppression (vs Other)	38.8
Satisfaction with supervisor	
Very satisfied (vs Less than very satisfied)	33.7
Years of experience	
5+ years (vs <5 years)	55.9
Practice Level	
Paramedic (vs EMT – Basic)	33.8
Area of residence or area served	
Rural (vs Non-rural)	51.5
Number of EMS organizational employers	
1 (vs 2+)	67.5
Primary role	
Clinician (vs Fire-suppression)	82.7

Table 2 describes the percent agreement or “yes” responses to the survey questions. Forty-three percent agreed that a pandemic flu emergency would occur in the community they serve, and 66% agreed that a pandemic flu emergency would have severe public health consequences. Ninety-three percent would be willing to report to work, if required, and 88% would be willing to report to work if asked, but not required. The conditional willingness-to-report questions ranged from 92–97% agreement levels. However, if there was a possibility for disease transmission to family members, the willingness-to-report rate was only 48%. Regarding personal emergency preparations, 51% had a family communication plan and 33–38% had other preparations. With respect to volunteering in other communities, 73% would volunteer for a serious situation, 80% would volunteer if the probability of becoming ill was low, 37% would volunteer if the probability of becoming ill was high, and 50% would volunteer if they were required to move for a week or more.

**Table 2:** Survey responses of EMS clinical service providers (weighted).

Attitudes and Beliefs Regarding Pandemic Flu Emergency	% Agree
Likelihood of pandemic flu occurring	42.6
Severity of consequences of pandemic flu	65.9
Likelihood of being asked to report to duty	83.2
If required: willing to report during pandemic flu emergency	93.1
If asked but not required: willing to report during pandemic flu emergency	88.1
Knowledgeable about public health impact	83.9
Awareness of role-specific responsibilities	78.3
Have skills for role-specific responsibilities	91.6
Psychologically prepared	92.9
Able to safely get to work	95.2
Confident in personal safety at work	85.6
Able to perform duties (Self-Efficacy)	93.5
Family is prepared to function in absence	87.5
EMS agency is able to provide timely information	82.1
Able to address public's questions	78.9
Importance of one's role in the EMS agency's overall response	87.8
Need for pre-event preparation and training	90.8
Need for psychological support during the event	70.9
Need for post-event psychological support	72.6
High impact of one's response (Response Efficacy)	87.4
**Would report to work if I:**	**% Probably or definitely yes**
Were guaranteed a pandemic flu vaccine	96.1
Were guaranteed vaccine for all my family members	95.6
Were guaranteed antiviral medicine if had unprotected exposure to ill patient	97.1
Were guaranteed medicine to prevent infection daily regardless of known exposure	95.5
Were guaranteed quarantined place for care (rather than home) if became ill	92.8
Were guaranteed priority for antiviral medicine if became ill and medicine in short supply	92.2
Thought people I live with might get flu from me	47.5
**Would report to pandemic flu emergency in another community:**	**% Probably or definitely yes**
If their problem was more serious than in own community	72.8
If their problem was less serious than in own community	46.2
If the other community did not take appropriate preventative measures	49.9
If the probability of becoming ill was low	79.9
If the probability of becoming ill was high	36.8
If it required moving for a week or more	49.9
**Additional questions**	**% Yes**
EMS agency provided preparation and training for pandemic flu emergency	39.1
Have family members living with/near me who rely on me for support	63.1
Have disaster supply kit in home	33.2
Have portable emergency supplies at home to take in case need to leave quickly	37.9
Have family communication plan in case of separation	50.8
Have family plan regarding place to meet if home is destroyed	35.3
Have family emergency fire or other drills at home	35.0
Received flu shot in past 12 months	59.1
	**% “A great deal” or “A moderate amount”**
Knowledge about pandemic flu	50.9

When EMS personnel knew their responsibilities in a pandemic influenza emergency, they were more likely than not to be willing to report to the emergency if required or if asked but not required [unadjusted OR(95%CI): 2.3 (1.03, 5.07) and 2.3 (1.21, 4.27), respectively]. Being prepared to perform their responsibilities increased the unadjusted odds (95%CI) for willingness to report if required or if asked but not required to 4.3 (1.71, 10.92) and 6.2 (2.86, 13.40) respectively. Perceiving that one's response role is important increased the unadjusted odds (95%CI) for willingness to report if required or if asked but not required to 5.3 (2.34, 11.90) and 6.5 (3.32, 12.64), respectively. These attitudes and beliefs were not related to willingness to report if there was a potential for disease transmission to family members.

When considering EMS professional characteristics that might predict willingness to report, those with self-perceived knowledge about the public health impacts of pandemic influenza were more likely to report if asked but not required, than those without such self-perceived knowledge [OR(95%CI): 2.5 (1.02, 6.23)] (Table 3), adjusting for the other EMS professional characteristics. EMS workers who indicated high efficacy on the EPPM, were more likely to report if asked, but not required than their counterparts in the low threat/low efficacy category, adjusting for other characteristics [OR(95%CI): 3.7 (1.18, 11.72) for low threat/high efficacy and 4.7 (1.20, 18.30) for high threat/high efficacy]. With respect to willingness to report if there was a potential to transmit disease to family members, EMS workers who were in the high threat/high efficacy category were more likely to report than their counterparts in the low threat/low efficacy category [OR(95%CI): 2.2 (1.07, 4.44)], adjusting for other characteristics. None of the characteristics were related to willingness to report if required. An evaluation of whether finer categorizations of the characteristics would impact the results was performed, and no significant change in the strength or direction of the associations was observed.

**Table 3:** Weighted multivariate logistic regression analyses to evaluate the associations between willingness-to-report scenarios and EMS characteristics regarding a pandemic influenza emergency (adjusted for all characteristics).

		Willing to Report If Asked But Not Required	Willing to Report If Required	Willing to Report If Potential for Disease Transmission to Family Members
EMS Characteristic	Reference Category	Odds Ratio^a^ (95%CI)	Odds Ratio^a^ (95%CI)	Odds Ratio^a^ (95%CI)
Female	Male	0.97 (0.44,2.13)	1.02 (0.37, 2.82)	0.97 (0.56, 1.70)
Age: >35 years	≤35 year	1.45 (0.66, 3.22)	0.56 (0.17, 1.84)	1.16 (0.66, 2.03)
Highest degree: At least a bachelor's degree	Less than bachelor's degree	1.18 (0.58, 2.42)	0.99 (0.37, 2.64)	0.67 (0.39, 1.16)
Number of EMS organizational employers: 1	2+	1.33 (0.62, 2.86)	1.61 (0.58, 4.53)	0.63 (0.38, 1.04)
Primary role: clinician	Fire suppression	0.71 (0.24,2.09)	0.68 (0.15, 3.06)	1.57 (0.73, 3.38)
Type of organization to which they belong: Fire-based	Other	1.10 (0.48, 2.51)	1.60 (0.53, 4.80)	1.14 (0.62, 2.09)
Satisfaction with supervisor: Very satisfied	Less than very satisfied	1.14 (0.52, 2.53)	0.94 (0.37, 2.44)	0.65 (0.39, 1.10)
Years of experience: 5+	<5	0.85 (0.35, 2.05)	1.40 (0.43, 4.51)	1.14 (0.63, 2.05)
Practice Level: Paramedic	EMT – Basic	0.83 (0.34, 2.07)	1.50 (0.44, 5.17)	1.30 (0.74, 2.27)
Area of residence or area served: Non-rural	Rural	0.76 (0.34, 1.67)	0.66 (0.24, 1.79)	0.69 (0.41, 1.17)
Knowledgeable about public health impact: Agree	Disagree	2.52 (1.02, 6.23)	2.49 (0.77, 8.01)	1.62 (0.81, 3.26)
Knowledge about pandemic flu: A great deal or moderate amount	Little or very little knowledge	0.75 (0.35, 1.62)	0.85 (0.32, 2.92)	1.12 (0.67, 1.86)
Extended Parallel Process Model Threat/Efficacy Profile				
Low threat/high efficacy	Low threat/low efficacy	3.72 (1.18, 11.72)	3.36 (0.74, 15.35)	1.34 (0.68, 2.63)
High threat/low efficacy	Low threat/low efficacy	0.92 (0.42, 2.02)	0.93 (0.31, 2.78)	0.93 (0.49, 1.80)
High threat/high efficacy	Low threat/low efficacy	4.68 (1.20, 18.30)	5.09 (0.90, 28.75)	2.18 (1.07, 4.44)
Received flu shot in past 12 months: Yes	No/Don't Know	0.81 (0.38, 1.75)	0.85 (0.32, 2.28)	0.72 (0.44, 1.18)
Have disaster kit in home: Yes	No/Don't Know	1.34 (0.65, 2.77)	1.54 (0.59, 4.05)	1.70 (0.99, 2.88)
Have family communication plan in case of separation: Yes	No/Don't Know	1.30 (0.59, 2.86)	0.90 (0.34, 2.34)	1.04 (0.63, 1.71)

a Outcome response: Agree compared to Disagree (reference category).

None of the EMS professional characteristics in Table 4 were related to the respondents' EPPM threat profile, after adjusting for the other characteristics. However, the characteristics distinguishing those with a high EPPM efficacy score included females more likely than males [OR(95%CI): 2.0 (1.15, 3.30)], and those who perceived themselves as knowledgeable about public health impacts of pandemic influenza more likely than those without that self-perceived knowledge [OR(95%CI): 3.0 (1.42, 6.19)], after adjusting for the other characteristics. Provision of preparation and training for a pandemic influenza emergency by the EMS worker's agency was not related to whether the EMS worker received an influenza vaccination [OR(95%CI) = 1.29 (0.86, 1.94)].

**Table 4:** Weighted multivariate logistic regression analyses to evaluate the associations between the Extended Parallel Process Model (EPPM) threat and efficacy dimensions and EMS characteristics regarding a pandemic influenza emergency (adjusted for all characteristics).

		EPPM Threat^a^	EPPM Efficacy^a^
EMS Characteristic	Reference Category	Odds Ratio^a^ (95%CI)	Odds Ratio^a^ (95%CI)
Female	Male	0.84 (0.49, 1.41)	1.95 (1.15, 3.30)
Age: >35 years	≤35 year	0.60 (0.35, 1.02)	1.10 (0.62, 1.94)
Highest degree: At least a bachelor's degree	Less than bachelor's degree	1.04 (0.62, 1.73)	0.79 (0.47, 1.33)
Number of EMS organizational employers: 1	2+	0.83 (0.50, 1.36)	0.78 (0.48, 1.27)
Primary role: clinician	Fire suppression	1.31 (0.61, 2.81)	0.71 (0.34, 1.51)
Type of organization to which they belong: Fire-based	Other	1.54 (0.86, 2.74)	0.75 (0.41, 1.37)
Satisfaction with supervisor: Very satisfied	Less than very satisfied	1.29 (0.79, 2.11)	1.54 (0.92, 2.59)
Years of experience: 5+	<5	1.10 (0.62, 1.95)	0.91 (0.50, 1.66)
Practice Level: Paramedic	EMT – Basic	1.15 (0.66, 2.01)	0.62 (0.36, 1.08)
Area of residence or area served: Non-rural	Rural	1.15 (0.70, 1.90)	1.12 (0.67, 1.88)
Knowledgeable about public health impact: Agree	Disagree	1.28 (0.66. 2.47)	2.96 (1.42, 6.19)
Knowledge about pandemic flu: A great deal or moderate amount	Little or very little knowledge	0.93 (0.57, 1.52)	1.02 (0.62, 1.70)
Received flu shot in past 12 months: Yes	No/Don't Know	0.90 (0.56, 1.46)	1.03 (0.64, 1.68)
Have disaster kit in home: Yes	No/Don't Know	0.84 (0.49, 1.42)	1.02 (0.59, 1.76)
Have family communication plan in case of separation: Yes	No/Don't Know	1.15 (0.70, 1.89)	1.41 (0.86, 2.32)

a Outcome response: High vs Low (reference category).

The attitudes and beliefs distinguishing an EMS worker's response willingness if asked but not required (Table 5) included: being prepared to perform their responsibilities in a pandemic influenza emergency [OR(95%CI) = 3.8 (1.41, 10.40)], and confidence about safety at work [OR(95%CI) = 3.3 (1.40, 7.67)], adjusting for the other attitudes and beliefs. The belief distinguishing an EMS worker's willingness to report if required (Table 5) was confidence about their safety at work [OR(95%CI) = 3.5 (1.28, 9.62)], adjusting for the other attitudes and beliefs. Generally census regions did not distinguish willingness to respond and other attitudes and beliefs, adjusting for EMS professional characteristics.

**Table 5:** Weighted multivariate logistic regression analyses to evaluate the associations between willingness-to-report scenarios and selected attitudes and beliefs regarding a pandemic influenza emergency (adjusted for other beliefs).

	Willing to Report If Asked But Not Required	Willing to Report If Required
Attitudes and Beliefs^a^	Odds Ratio^a^ (95%CI)	Odds Ratio^a^ (95%CI)
Awareness of role-specific responsibilities	0.90 (0.38, 2.11)	1.02 (0.35, 3.00)
Have skills for role-specific responsibilities	3.83 (1.41, 10.40)	2.42 (0.76, 7.71)
Confident in personal safety at work	3.28 (1.40, 7.67)	3.51 (1.28, 9.62)
Family is prepared to function in absence	1.91 (0.71, 5.10)	1.00 (0.29, 3.45)
EMS agency is able to provide timely information	1.67 (0.70, 4.00)	1.32 (0.39, 4.40)
Need for psychological support during the event	0.61 (0.18, 2.09)	0.61 (0.12, 3.02)
Need for post-event psychological support	2.22 (0.63, 7.90)	4.63 (0.97, 22.10)
EMS provided preparation and training for pandemic flu emergency	1.42 (0.66, 3.05)	2.30 (0.77, 6.91)

a Outcome response: Agree compared to Disagree (reference category).

Six scenarios were presented regarding the EMS worker's willingness to respond to a pandemic influenza emergency in another community (Table 6). After adjusting for the other attitudes and beliefs, confidence in personal safety at work would lead an EMS worker to be more willing to volunteer in another community if the emergency was more serious than in their community [OR(95%CI) = 2.3 (1.19, 4.48)]; if the emergency was less serious than in their community [OR(95%CI) = 2.4 (1.18, 5.00)]; and if the probability of becoming ill is high [OR(95%CI) = 2.1 (1.04, 4.23)]. This confidence increased the odds slightly more for willingness to volunteer if the other community had taken no preventive measures [OR(95%CI) = 3.8 (1.98, 7.37)] or if the probability of becoming ill was low [OR(95%CI) = 3.5 (1.51, 8.32)].

**Table 6:** Weighted multivariate logistic regression analyses to evaluate the associations between willingness-to-report scenarios in another community and selected attitudes and beliefs regarding a pandemic influenza emergency (adjusted for other beliefs).

	Willing to Report If More Serious than in Own Community	Willing to Report If Less Serious than in Own Community	Willing to Report If Other Community Did Not Take Appropriate Prevention Measures
Attitudes and Beliefs^a^	Odds Ratio^a^ (95%CI)	Odds Ratio^a^ (95%CI)	Odds Ratio^a^ (95%CI)
Awareness of role-specific responsibilities	0.81 (0.45, 1.45)	0.83 (0.47, 1.47)	1.33 (0.76, 2.35)
Have skills for role-specific responsibilities	1.92 (0.76, 4.83)	1.32 (0.50, 3.53)	0.97 (0.38, 2.48)
Confident in personal safety at work	2.31 (1.19, 4.48)	2.4 (1.18, 5.00)	3.82 (1.98, 7.37)
Family is prepared to function in absence	2.35 (1.16, 4.76)	2.31 (1.09, 4.90)	2.22 (1.10, 4.48)
EMS agency is able to provide timely information	1.28 (0.68, 2.40)	1.34 (0.73, 2.47)	0.88 (0.48, 1.61)
Need for psychological support during the event	0.75 (0.31, 1.81)	1.50 (0.64, 3.52)	0.49 (0.23, 1.05)
Need for post-event psychological support	1.57 (0.63, 3.93)	0.75 (0.31, 1.80)	1.42 (0.64, 3.12)
EMS provided preparation and training for pandemic flu emergency	1.04 (0.63, 1.71)	1.36 (0.88, 2.11)	1.44 (0.92, 2.24)
	**Willing to Report If High Probability of Becoming Ill**	**Willing to Report If Low Probability of Becoming Ill**	**Willing to Report For Move of Week or Longer**
	**Odds Ratio** a **(95%CI)**	**Odds Ratio** a **(95%CI)**	**Odds Ratio** a **(95%CI)**
Awareness of role-specific responsibilities	0.78 (0.40, 1.54)	0.84 (0.46, 1.54)	1.20 (0.69, 2.07)
Have skills for role-specific responsibilities	2.16 (0.81, 5.80)	1.86 (0.65, 5.28)	1.59 (0.64, 3.96)
Confident in personal safety at work	2.09 (1.04, 4.23)	3.54 (1.51, 8.32)	1.53 (0.83, 2.83)
Family is prepared to function in absence	1.45 (0.61, 3.44)	3.81 (1.63, 8.92)	3.00 (1.52, 5.90)
EMS agency is able to provide timely information	1.65 (0.82, 3.32)	0.77 (0.41, 1.47)	1.50 (0.85, 2.66)
Need for psychological support during the event	0.76 (0.25, 2.32)	0.64 (0.30, 1.34)	0.67 (0.29, 1.55)
Need for post-event psychological support	1.66 (0.53, 5.27)	1.32 (0.61, 2.84)	1.30 (0.56, 3.04)
EMS provided preparation and training for pandemic flu emergency	1.23 (0.71, 2.12)	1.69 (1.09, 2.64)	0.93 (0.60, 1.44)

a Outcome response: Agree compared to Disagree (reference category).

When family was prepared to function in their absence, an EMS worker was also more willing to volunteer in another community if the emergency was more serious than in their community [OR(95%CI) = 2.3 (1.16, 4.76)]; if the emergency was less serious than in their community [OR(95%CI) = 2.3 (1.09, 4.90)]; and if the other community had taken no preventative measures [OR(95%CI) = 2.2 (1.10, 4.48)]; if the probability of becoming ill was low [OR(95%CI) = 3.8 (1.63, 8.92)]; and if moving for a week or more was required [OR(95%CI) = 3.0 (1.52, 5.90)], after adjusting for the other attitudes/beliefs.

EMS workers who received an influenza immunization in the past 12 months were compared with those who did not, with respect to 36 demographic and other individual and work environment characteristics for which data were available (Table S1). EMTs and Paramedics who were 36 years of age and older were significantly more likely to have been vaccinated than younger EMS personnel (64.5% vs. 50.7%, p = .0057). There was also a tendency for EMS workers who worked for a fire-based service to avoid vaccinations (54.7% vs. 64.3%, p = .0691) and for rural EMS personnel to avoid vaccinations (56.2% vs. 64.8%, p = .0735). EMS workers with family members living with or near them who relied on them for support were more likely to have been vaccinated in the past 12 months (62.6% vs. 53.4%, p = .0700). However, with the exception of age, none of these relationships were statistically significant.

## Discussion

Response willingness is an essential ingredient of healthcare system capacity across the all-hazards spectrum. Our pandemic influenza-focused results in this study reinforce a critical finding from previous LEADS-based research on U.S. EMS workers' response attitudes toward non-pandemic influenza scenarios – namely, that the willingness of these workers to fulfill response roles during large-scale public health crises cannot be universally assumed [1]. With minimal regional variation, overall 12% of the workers in our study would not voluntarily report to duty in a pandemic influenza emergency when asked, and 7% of the workers would not report to duty even if required.

Of concern, our study revealed that the majority (52%) of EMS workers would stay home if a risk of disease transmission to family existed. This was the case irrespective of knowing one's role or recognizing its importance, contrary to previous research that showed that the perception of the importance of one's role in the agency's response and understanding one's role-specific response requirements were among the leading predictors of willingness to respond for local health department workers [3]. As such disease transmission risk always exists in an influenza pandemic, this finding has significant operational implications not only for EMS, but also for the overall healthcare system response infrastructure as a consequence. Further, mobilizing EMS personnel to particularly hard-hit communities during a pandemic will not be easy. We found that 20% of the respondents would be reluctant to do so, even if they thought the probability of becoming ill was low. Since an influenza pandemic can be expected to exert disparate surge capacity demands on different communities at varying times, this finding represents a substantial EMS challenge to be tackled.

During the global emergence of a novel influenza strain, already spreading rapidly in the US throughout the survey window and declared a pandemic by the WHO on 11 June 2009, less than half (43%) agreed that this event would occur in the community they serve, and only 66% agreed that a pandemic influenza event would have severe public health consequences. These responses may likely have reflected that the inevitability of the strain's dissemination and scope of impact had not been fully grasped by the public at that point.

Given previously-recognized EMS infrastructure challenges [16] and amidst a highly contagious strain, our study's findings present a problematic landscape for prehospital healthcare system capacity in the current pandemic. However, our findings also simultaneously highlight opportunities for impactful interventions to boost pandemic influenza response willingness among this cadre of first responders. Consonant with past research on EMS workers' response willingness in terrorism scenarios [1], [20], our findings reveal the importance of hazard-specific response education: knowing one's role in a pandemic more than doubled an EMS worker's likelihood of voluntarily reporting (unadjusted OR = 2.3), while recognizing the importance of one's role increased such willingness more than six-fold (unadjusted OR = 6.5).

Additionally, our results indicate that emphasis on personal and family preparedness planning is strongly advisable in the context of pandemic influenza education for EMS workers. If the family is prepared to function in their absence, EMS personnel were more than twice as willing to mobilize to another, more severely affected community (OR = 2.3), after adjusting for other attitudes and beliefs. These findings reinforce those of a UK study showing healthcare workers with caring responsibilities to be significantly less likely to report to work than those without dependents, and a study in the US in which over 20% of workers agreed that personnel without children should be the primary responders in a pandemic [12], [21]. Preparing families of healthcare workers, including EMS workers, will be critical in ensuring an adequate public health response in a pandemic emergency. Instilling confidence in occupational safety in an influenza pandemic also appears critically important for this healthcare provider cohort. EMS workers who were confident in their work environment safety in an influenza pandemic were more than three times as likely to voluntarily report to duty in such an event (OR = 3.3), and were more than twice as likely to be willing to mobilize for response to another more severely affected community (OR = 2.3).

Importantly, our findings also suggest the relevance of the Extended Parallel Process Model (EPPM) to inform educational efforts that explicitly address EMS workers' perceptions of threat and efficacy toward pandemic influenza response. Those fitting a “concerned and confident” (high threat/high efficacy) EPPM profile were more than four times as likely (OR = 4.7) to be willing to report to work if asked but not required, after adjusting for other covariates. This highest level of willingness among the “high threat/high efficacy” group is consistent with a pattern observed in earlier EPPM survey-based research we conducted on local public health workers' willingness to respond in an influenza pandemic [4]. Moreover, consistent with that previous research, we found that perceived efficacy carried substantial weight among EMS providers: those fitting a “low threat/high efficacy” profile were still more than three times as likely (OR = 3.7) to be willing to respond, after adjusting for other covariates. Nationally to date, healthcare workforce emergency preparedness trainings have focused nearly exclusively on cognitive (ability-focused) rather than affective (willingness-focused) domains of response. However, our findings highlight the need for enhanced attention to pandemic response-related attitudes in the context of EMS workforce trainings, and point to the EPPM as a potentially useful framework for informing these offerings.

Despite alternative terminology used in risk perception modeling, the identification of a simultaneous evaluation of affective and analytic processes in risk perception reinforces the use of the EPPM as a model of choice in informing pandemic-related educational efforts [22]. One risk perception model describes the perceptual characterization of risk through two main axes: risk familiarity (unknown risk is perceived as higher risk) and level of dread associated with risk [23]. Similar to the EPPM in which threat and efficacy are simultaneously evaluated, this risk perception model identifies a parallel interplay of logical and emotional processes [22]. In the context of the risk-benefit paradigm, an individual decision is based on both thoughts and feelings. If the outcome of the decision is perceived to be emotionally positive, the risk will be viewed as low and the benefit as high, increasing the likelihood of the decision (action) [22], [24]. The critical importance of affective evaluations in this risk perception model underscores the relevance of attitudinal interventions as informed by the EPPM, for pandemic willingness-related response trainings for EMS personnel.

We noted that 41 percent of the EMTs and Paramedics who responded to this survey did not receive an influenza immunization in the last 12 months. Since it seems likely that the same types of individuals who do not receive seasonal flu vaccination may also be negatively predisposed to pandemic H1N1 2009 influenza A vaccinations, we compared the characteristics of EMS personnel who received flu vaccinations with those who did not. Other than age (older EMTs and Paramedics were more likely to get vaccinated than younger ones), we were unable to identify any significant relationships between individual characteristics and vaccination propensities.

We also noted that 52 percent of the EMTs are in rural areas (that is, they do most of their EMT work in areas or towns with fewer than 25,000 people). These data are consistent with previous findings in the literature [25]. However, as call volumes in rural areas are much lower than in other areas, this datum should not be interpreted to mean that 52% of the nation's EMS services are provided in rural areas.

Certain limitations to this study need to be acknowledged. First, the 49% survey response rate, while comparable to that of earlier research on willingness of U.S. EMS personnel to respond to terrorist incidents [1], may have introduced the potential for non-response bias in the current study. However, a high level of survey responder versus non-responder demographic similarity has been noted in previous LEADS cohort analyses [25], [26]. A second noteworthy limitation of the current study is that responses to a survey of this type may not necessarily predict actual behavior. Most studies to date linking healthcare workers' intention to their behavior have recognized methodological flaws including lack of experimental design, poor methods in measuring behavior, poor matching of the context of the intention with the behavior measured, and poor reporting of studies [27], [28]. Despite both limited quality and quantity of literature, a theoretical framework developed through a meta-analysis of 78 studies examining the effectiveness of social cognitive theories in explaining the relationship between intention and behavior presents intention – influenced by belief about consequences, social influences, moral norms, role and identity and characteristic – to be one of the most proximal causation factors to actual behavior. However, the accuracy of this behavior prediction decreases as the complexity of a situation and the number of modulating factors increases.. Although this meta-analysis did not look at the use of the EPPM as a method of predicting behavior or informing educational intervention, it highlights the ability of models, specifically the Theory of Planned Behavior (TPB), to predict behavior in healthcare workers [28]. In the context of a pandemic, it is noteworthy that non-healthcare workers' actualization of their intended preventive and avoidant behaviors has been found to increase if there is a high level of associated anxiety, perceived susceptibility to or severity of disease, and perceived effectiveness of the behavior (response efficacy) [29]. All of these factors would be heightened for EMS workers in the event of a pandemic, suggesting that their intentions may also correspond to their actual behavior in this situational context.

The LEADS snapshot survey for this study was developed in January 2009, prior to the earliest case identifications of H1N1 2009 influenza A virus; as a consequence, the survey did not explicitly refer to “pandemic H1N1 2009 influenza A” or “swine flu” in the context of its pandemic influenza questions. Although the survey was launched on May 15, 2009, the World Health Organization did not declare a pandemic until June 11, 2009, which was part of the survey window. However, it should be noted that as of the survey launch date, a total of 34 countries had officially reported 7,520 cases of H1N1 2009 influenza A infection to the World Health Organization, including 4,298 laboratory confirmed human cases and three deaths in the United States [30]; additionally, on April 29, 2009, approximately two weeks prior to the survey launch date, the World Health Organization had already raised its pandemic alert level to Phase 5, signaling a pandemic was imminent [31]. Finally, some of the demographic data used in the analyses came from the 2008 LEADS Survey, which was administered six months prior to this survey. Analyses of past LEADS Surveys indicate little change in these data (other than satisfaction with one's supervisor) over a 12-month period.

In conclusion, our study reveals the importance of underlying attitudes and beliefs that may substantially hinder willingness to report to duty among EMS workers in a global public health emergency. Given the “all available hands on deck” nature of pandemic influenza response, the results of our survey indicate that insufficient attention to these attitudinal domains of response can have a significantly detrimental impact on prehospital providers' capacity to meet surge challenges. Explicit attention to the realms of perceived threat and efficacy within EMS readiness trainings may serve to help overcome these identified attitudinal barriers, yielding an EMS workforce that is not only able to respond to a pandemic threat in requisite numbers, but willing to do so.

## Supporting Information

Table S1 Weighted chi-square analyses to evaluate the associations between EMS clinical service provider characteristics and influenza immunization in last 12 months.
(0.11 MB DOC)
