# Supplementary material for: Gauging U.S. Emergency Medical Services Workers' Willingness to Respond to Pandemic Influenza Using a Threat- and Efficacy-Based Assessment Framework
Source: PLoS One. 2010 Mar 24;5(3):e9856. doi: 10.1371/journal.pone.0009856 (PMC2844432; doi:10.1371/journal.pone.0009856)
Supplement: Table S1 — Weighted chi-square analyses to evaluate the associations between EMS clinical service provider characteristics and influenza immunization in last 12 months. (0.11 MB DOC) [file pone.0009856.s001.doc]

**Table S1. Weighted chi-square analyses to evaluate the associations between EMS clinical service provider characteristics and** influenza immunization in last 12 months

| **Characteristic** | **Category** | **% Vaccinated** | **p-value** |
| --- | --- | --- | --- |
| Gender | Male | 58.40% |  |
|  | Female | 59.50% | 0.849 |
|  |  |  |  |
| Age (years) | 36 & over | 64.50% |  |
|  | 18 - 35 | 50.70% | 0.006 |
|  |  |  |  |
| Education | At least bachelor’s degree | 63.80% |  |
|  | Less than bachelor’s degree | 57.60% | 0.236 |
|  |  |  |  |
| Number of EMS employers | 1 | 58.30% |  |
|  | 2 or more | 61.20% | 0.568 |
|  |  |  |  |
| Employer | Fire-based service | 54.70% |  |
|  | Other | 64.30% | 0.069 |
|  |  |  |  |
| Satisfaction with immediate supervisor | Less than very satisfied | 59.20% |  |
|  | Very satisfied | 63.10% | 0.447 |
|  |  |  |  |
| Experience (years) | 0 - 4 | 56.70% |  |
|  | 5 or more | 61.50% | 0.33 |
|  |  |  |  |
| Practice Level | Basic | 59.70% |  |
|  | Paramedic | 58.40% | 0.763 |
|  |  |  |  |
| Area served or area of residence | Not rural | 64.80% |  |
|  | Rural | 56.20% | 0.074 |
|  |  |  |  |
| Test Score (on pandemic influenza knowledge items) | High | 59.20% |  |
|  | Low | 59.30% | 0.987 |
|  |  |  |  |
| Self-Assessed Pandemic Influenza Knowledge | Great or moderate amount | 60.10% |  |
|  | Little or very little amount | 58.20% | 0.692 |
|  |  |  |  |
| Threat (Extended Parallel Process Model) | High | 58.80% |  |
|  | Low | 59.00% | 0.963 |
|  |  |  |  |
| Efficacy (Extended Parallel Process Model) | High | 60.00% |  |
|  | Low | 58.40% | 0.731 |
|  |  |  |  |
| Extended Parallel Process Model Threat/Efficacy Profile | High threat/high efficacy | 61.10% |  |
|  | High threat/low efficacy | 56.60% |  |
|  | Low threat/low efficacy | 59.10% |  |
|  | Lot threat/low efficacy | 58.50% | 0.933 |
|  |  |  |  |
| Disaster kit at home? | No | 57.80% |  |
|  | Yes | 61.80% | 0.436 |
|  |  |  |  |
| Emergency communication plan at home? | No | 58.70% |  |
|  | Yes | 59.60% | 0.851 |
|  |  |  |  |
| Receive pandemic influenza training? | No | 59.10% |  |
|  | Yes | 62.70% | 0.479 |
|  |  |  |  |
| Proximate family members who rely on you for support? | No | 53.40% |  |
|  | Yes | 62.60% | 0.07 |
|  |  |  |  |
| Seat belt use (for EMS work)? | Always | 60.00% |  |
|  | Not always | 54.00% | 0.352 |
|  |  |  |  |
| Seat belt use (non-work)? | Always | 58.90% |  |
|  | Not always | 64.00% | 0.402 |
|  |  |  |  |
| Alcohol in last 30 days? | No | 57.40% |  |
|  | Yes | 60.10% | 0.603 |
|  |  |  |  |
| Number alcoholic drinks per drinking day | 4 or less | 60.10% |  |
|  | 5 or more | 68.60% | 0.433 |
|  |  |  |  |
| Number days with 5 or more alcoholic drinks | 1 or more | 61.30% |  |
|  | None | 60.40% | 0.881 |
|  |  |  |  |
| Smoke 100+ cigarettes in lifetime? | No | 57.10% |  |
|  | Yes | 64.10% | 0.183 |
|  |  |  |  |
| Daily smoker? | No | 66.50% |  |
|  | Yes | 58.80% | 0.409 |
|  |  |  |  |
| If smoker, did you try to quit in last year? | No | 58.10% |  |
|  | Yes | 58.90% | 0.854 |
|  |  |  |  |
| Trained in Air Purifying Respirator (APR) use? | Yes | 61.50% |  |
|  | No/Don’t know | 57.90% | 0.473 |
|  |  |  |  |
| Trained in personal protective equipment use? | Yes | 61.90% |  |
|  | No | 53.10% | 0.088 |
|  |  |  |  |
| Trained in Biological/Chemical/ Nuclear (BCN) hazard exposure? | Yes | 57.20% |  |
|  | No | 62.70% | 0.274 |
|  |  |  |  |
| Training in incendiary/explosive exposure? | Yes | 56.80% |  |
|  | No | 61.30% | 0.35 |
|  |  |  |  |
| Training in structural collapse? | Yes | 54.50% |  |
|  | No | 62.60% | 0.095 |
|  |  |  |  |
| Training in incident command/management system? | Yes | 58.20% |  |
|  | No/Don’t know | 62.10% | 0.493 |
|  |  |  |  |
| Participate in multi- agency biological, chemical, or nuclear (BCN) drills? | Yes | 62.80% |  |
|  | No | 57.50% | 0.33 |
|  |  |  |  |
| Participate in multi-agency explosives drill? | Yes | 57.90% |  |
|  | No | 59.40% | 0.802 |
|  |  |  |  |
| Does agency have emergency scene ID procedures? | Yes | 63.00% |  |
|  | No/Don’t know | 55.80% | 0.135 |
|  |  |  |  |
| Does agency maintain antibiotic cache? | Yes | 67.30% |  |
|  | No/Don’t know | 56.60% | 0.058 |
